# Beyond the hype: using AI, big data, wearable devices, and the internet of things for high-throughput livestock phenotyping

**DOI:** 10.1093/bfgp/elae032

**Published:** 2024-08-19

**Authors:** Tomas Klingström, Emelie Zonabend König, Avhashoni Agnes Zwane

**Affiliations:** Department of Animal Biosciences, Swedish University of Agricultural Sciences, Uppsala, Sweden; SLU Global, Swedish University of Agricultural Sciences, Uppsala, Sweden; Department of Biochemistry, Genetics and Microbiology, University of Pretoria, Pretoria, South Africa

**Keywords:** phenotyping, livestock, high-throughput, artificial intelligence, genetics, internet of things

## Abstract

Phenotyping of animals is a routine task in agriculture which can provide large datasets for the functional annotation of genomes. Using the livestock farming sector to study complex traits enables genetics researchers to fully benefit from the digital transformation of society as economies of scale substantially reduces the cost of phenotyping animals on farms. In the agricultural sector genomics has transitioned towards a model of ‘Genomics without the genes’ as a large proportion of the genetic variation in animals can be modelled using the infinitesimal model for genomic breeding valuations. Combined with third generation sequencing creating pan-genomes for livestock the digital infrastructure for trait collection and precision farming provides a unique opportunity for high-throughput phenotyping and the study of complex traits in a controlled environment. The emphasis on cost efficient data collection mean that mobile phones and computers have become ubiquitous for cost-efficient large-scale data collection but that the majority of the recorded traits can still be recorded manually with limited training or tools. This is especially valuable in low- and middle income countries and in settings where indigenous breeds are kept at farms preserving more traditional farming methods. Digitalization is therefore an important enabler for high-throughput phenotyping for smaller livestock herds with limited technology investments as well as large-scale commercial operations. It is demanding and challenging for individual researchers to keep up with the opportunities created by the rapid advances in digitalization for livestock farming and how it can be used by researchers with or without a specialization in livestock. This review provides an overview of the current status of key enabling technologies for precision livestock farming applicable for the functional annotation of genomes.

## Introduction

One of the ‘grand challenges’ in modern biology is to understand the genetic basis of phenotypic diversity within and among species [1]. The livestock sector can provide an advanced infrastructure for high throughput genotyping and phenotyping of animals. Although big datasets from livestock have been collected since the beginning of the 20th century the rise of precision farming and overall digitalization of society mean that the amount of data recorded about animals and their environment on farms is growing at a rapid pace [2]. Precision livestock farming can here be defined as a process where data from the sensors generate feedback to the controller so that he or she can make informed decisions based on a decision model in order to generate a set of desired responses in the herd being managed [3]. For breeding purposes industrial scale genotyping of livestock is performed simultaneously [4] which can provide access to 45 k Single-nucleotide polymorphism (SNP) genotyping at a cost of <30 euro per animal if performed in collaboration with breeding organizations [5]. This article will give an overview of how farming practices in a digitalized society can enable large-scale studies on phenomics in high as well as low-income settings as digital technology has become commonplace on a global scale.

### Scope

This narrative review will focus on four key areas for the utilization of livestock farming data for high-throughput phenotyping of animals:

- The types of measurements used in farming applicable for phenotyping in genetics research.
- The development of new sensors for the digitalization of on-farm equipment generating data which are currently collected in herd management systems on farms to support on-farm operations [6].
- How the digitalization of agriculture and precision livestock farming generate contextual information about farm animals and their environment.
- Describing the role of breeding organizations and breeding programs which may be utilized for high-throughput phenotyping.

The aim of this review is to narratively describe how technology and precision livestock farming enables the measurements necessary for high-throughput phenotyping. As a technology oriented review this scope excludes the process of converting a set of measurements into an observed phenotype or the calculation of breeding values.

### Phenotype measurements in agriculture

The observation and quantification of genetic traits is a key activity when selecting animals for breeding. In this review we highlight five different kinds of measurements currently in use for livestock breeding and precision livestock farming.

- One time measurements – a measurement made once for persistent characteristics such as polledness.
- Longitudinal data – repeated measurements giving a single value such milk yield used in milk recordings [7].
- Behavioural measurements measuring the displayed behaviour of an individual [8].
- Endophenotypes or intermediate phenotypes using biomarkers which may be more accessible or stable than measured phenotypes for example used when studying genetic susceptibility to a metabolic disorder.
- Contextual phenotyping where measuring the environmental context of an animal can enable measurements of traits related to phenotype plasticity [9] and robustness [10].

From historical records easily recorded characteristics concerning the exterior of an animal has been very important to define a breed. The physical form of an animal is often correlated with other adaptions to the local environment or farming practices. It is therefore often possible to use historical records to identify phenotypes distinct for specific geographical regions or breeds which can provide valuable information regarding the complex interplay of artificial selection, population admixture, inbreeding and genetic drift for genetics research [11]. Longitudinal data collection requires more persistent efforts and herd books and milk recording organizations became prominent in the early 20th century [7]. Data collection efforts enabled organizations to track progress over time and supported the organization of data in such ways that more complex traits such as total milk yield per lactation could be estimated. Simple collections have evolved to modern day breeding programs where a large number of traits related to productivity, health, and other production factors are being measured which is further described in the section ‘Data recording for breeding and advisory organizations’.

An area receiving increased attention in livestock research is the automated collection of behavioural information. Temperament is often used as a composed trait for selection of animals in breeding programs but usually relies on personal observations which do not generally work well to enable the identification of separate genetic traits [12, 13].With improved animal tracking within farms high-throughput phenotyping of behaviour has become more viable. This tracking can take the form of movement sensors [14] or gate passages using farm equipment such as autonomous milking systems and smart gates [15] as well as by using novel solutions relying on machine vision [16, 17] to visually track animal behaviour which makes it possible to study even complex social behaviour in a production environment [14, 18].

Endophenotypes are commonplace in medicine as samples are taken and analysed in the healthcare system. For specific applications a similar approach can be taken for livestock and some farms have invested in compact on-farm labs like the Herd Navigator [19]. Such machines are expensive but make it possible to measure hormones and metabolites such as progesterone to detect heat, lactate dehydrogenase to detect early mastitis, betahydroxybutyrate for ketosis and urea to adjust protein intake for animals.

Contextual information is another area where digitalization and automation are drastically reducing costs of data collection. Open data strategies in the public sector and funding for ambitious data collection projects such as the Sentinel-2 satellites mean that substantial amounts of data regarding weather, biomass growth and water availability are now available on a weekly basis down to a resolution of 10 × 10 m [20]. These data can be combined with data from other sources such as feed producers, fixed environmental sensors and geospatial data from satellites and/or drones, this have created an emerging field of big data within precision livestock farming using remote sensing for both feed production and herd monitoring [21]. When combined with animal data this information makes it possible to measure traits related to phenotypic plasticity [8, 22] or robustness [10, 23] as the expressed phenotype can be put into a context of the environmental conditions which have shaped the animal.

### Economic considerations of phenotyping in livestock farming

**Figure 1:**
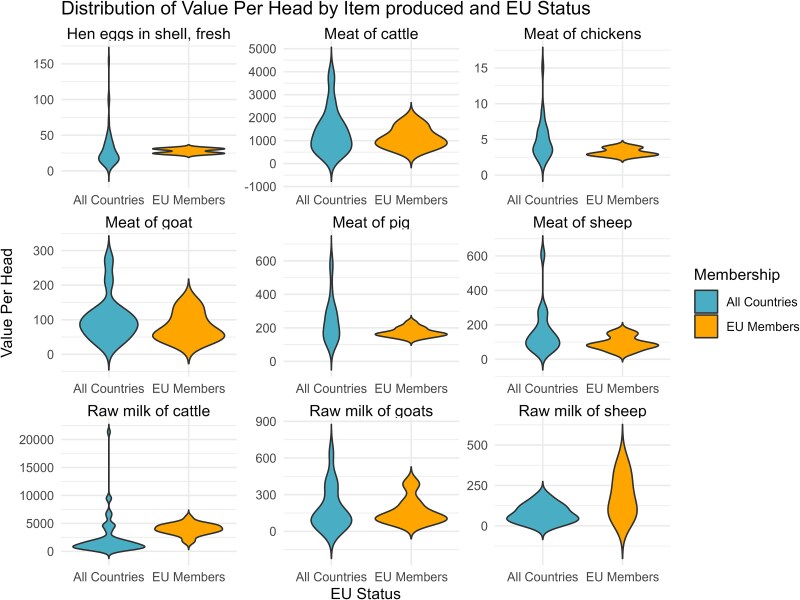
Violin plots of the food items produced by species included in the review. As indicated by table 1 and 2 the variation of annual production value is lower among European Union members but median production values are similar (FAO. FAOSTAT. License: CC BY-NC-SA 3.0 IGO. Extracted from: https://www.fao.org/faostat/en/#data. Data of Access: 2024-06-27).

The economic value of an individual is directly related to the amount of money which can be invested in monitoring it. An estimate of global total output value of farmed animals based on the FAOSTAT database operated by the Food and Agriculture Organization of the United Nations [24] indicate that the global economic value of livestock outputs are dominated by the value that cattle outputs generate (34% in 2018), followed by chickens (21%), pigs (17%), aquaculture (14%), other livestock (12%), and sheep (2%) [25]. Using a similar approach to the methodology presented by Schrobback *et al.* the annual production value per producing animal can be calculated (supplementary material 1). The use of annual income per animal used in production is necessitated by a lack of data on culling rates and the way total population statistics are being aggregated on a species level rather than production system or type of output. Dairy from cattle generates the highest annual income per individual in production with a median of 1789 USD per head and year. Meat from cattle generates the second highest economic value with a median income of 1097 USD per head. In comparison a pig brings in a median value of 171 USD per head, sheep 102 USD per head, goat meat 87 USD per head, and chickens a median of 4 USD. Goats used for dairy bring in 121.8 USD per animal and year while sheep used for dairy are reported to generate an annual value of 83.1 USD during their productive years. To better display the variation in production value between countries, Fig. 1 provides a set of violin plots displaying the distribution of reported income per product in countries reporting data to FAOSTAT, Table 1 display the median value, standard deviation and number of reporting countries in total and Table 2 the same statistics but for the single European market. A link to the calculations and source data is available in Supplementary Material 1. It is important to note that this data describe revenue per producing animal and year and not the overall income or profitability of the animals. A dairy cow will join the productive population at ~2 years of age but will remain productive for several years, while a chicken for meat production will only live for 50–60 days. On a herd level this mean that individual tracking where a sensor is attached to an animal is more attractive for long-lived animals generating value over multiple years while fixed installations that remain in place as individuals are slaughtered and replaced become more attractive for short-lived production animals. This is also reflected in this review as cattle production with its high economic value and longevity of production animals make use of a wide variety of wearable devices or fixed installations with identification provided by a transponder or radio-frequency identification (RFID) tags worn by individual animals. Emerging fields such as video surveillance and sound monitoring will however have a higher value for high-throughput production of animals such as broiler chickens and pig farming as recording devices can be used for multiple generations of animals each year which can be combined with existing fixed installations and RFID-based identification [26].

**Table 1:** The median value of annual production (USD) per productive animal by item produced, standard deviation (SD), number of productive animals (slaughtered, milked or egg-laying during the year) and number of countries reporting data to FAOSTAT (FAO. FAOSTAT. License: CC BY-NC-SA 3.0 IGO).

Item	USD per head	SD	Nr of Animals (million)	Nr of Countries
Hen eggs in shell, fresh	22.8	24.9	5485.9	61
Meat of cattle with the bone, fresh or chilled	1096.8	829.4	50.6	38
Meat of chickens, fresh or chilled	4	2.7	21611.7	29
Meat of goat, fresh or chilled	87.1	60.3	35.6	20
Meat of pig with the bone, fresh or chilled	171.2	112.7	986.3	35
Meat of sheep, fresh or chilled	101.9	111.1	311	30
Raw milk of cattle	1788.6	2942.3	111	82
Raw milk of goats	121.8	156.9	33.2	24
Raw milk of sheep	83.1	98.4	65.6	18

Extracted from: https://www.fao.org/faostat/en/#data. Date of Access: 2024-06-27). The global data highlight a large variation in annual production value and the median value has therefore been highlighted over the mean value, see supplementary material 1 for a breakdown of data on the national level.

**Table 2:** The median value of annual production (USD) per productive animal by item produced, standard deviation (SD), number of productive animals (slaughtered, milked or egg-laying during the year) among the European Union members which report data to FAOSTAT (FAO. FAOSTAT. License: CC BY-NC-SA 3.0 IGO).

Item	USD per head	SD	NrAnimals (million)	NrCountries
Hen eggs in shell, fresh	27.8	4.6	5	2
Meat of cattle with the bone, fresh or chilled	1057.6	437.5	7.8	12
Meat of chickens, fresh or chilled	3	0.6	464.8	3
Meat of goat, fresh or chilled	57.2	41.9	1.4	5
Meat of pig with the bone, fresh or chilled	168.7	27.8	140.2	13
Meat of sheep, fresh or chilled	88.8	39.8	17.4	11
Raw milk of cattle	3871.4	1006.7	14.9	23
Raw milk of goats	118.2	111.4	4.7	7
Raw milk of sheep	153.2	131.9	16.6	6

Extracted from: https://www.fao.org/faostat/en/#data. Date of Access: 2024-06-27). The global data highlight a large variation in annual production value and the median value has therefore been highlighted over the mean value, see supplementary material 1 for a breakdown of data on the national level.

### Data recording for breeding and advisory organizations

For inclusion in a breeding program any trait measured must be heritable and of sufficient economic importance to justify the costs of measuring. To ensure that enough animals can be measured to accurately calculate genomic breeding values there must be a reference population of sufficient size to perform the calculations [27]. Relatively straightforward traits to measure are therefore preferred in data collection for breeding evaluations. Over time the scope of traits targeted for selection have moved away from being purely production oriented toward a more balanced breeding goal covering aspects such as health and fertility in addition to productivity traits [7]. The Nordic countries have long held a leading role in this process [7, 28] making the Nordic Total Merit Index a good example of the scope of such a modern breeding program with 83 different measurements being performed to calculate 16 different sub-indices. These sub-indices concern milk yield, growth, fertility, birth index, calving index, udder health, general health, claw health, frame, feet and legs, udder, milkability, temperament, longevity, youngstock survival and saved feed (see supplementary materials 2, Table 2 for a full list of measurements). For a more comprehensive overview of traits currently used in breeding programs and how they are measured, the International Bull Evaluation Service (Interbull) host a compiled list of traits used in various national genetic evaluations from member organizations (https://interbull.org/ib/geforms). A more historical perspective of breeding programs is provided by the review of Miglior *et al.* [7] and provides a valuable introduction to the field for geneticists looking to utilize data from livestock in their research.

International standardization of testing is a major challenge for breeding organizations as exemplified by the differences in procedures used in national genetic evaluations submitted to Interbull. Researchers with different specializations, or working in different regions, will therefore have to rely on different nomenclatures and standards when using production data for high-throughput phenotyping of animals. It is therefore important to connect phenotypes collected from animals in recordings and breeding programs with definitions from ontologies such as the animal trait ontology [29] and databases such as the Animal QTLdb [30] to link the applied animal science to knowledge structures used in genetics, molecular biology and the functional annotation of genomes.

Establishing sustainable animal recordings in low- or middle income countries have proven to be challenging as a lack of economically sustainable infrastructure makes it difficult to maintain records over long periods of time and return value of the recordings to farmers. Conceptually the challenges of establishing reliable animal recordings in low-or middle income countries can be described as a wicked problem [31] where the lack of organized recording makes it difficult to obtain a premium valuation for premium animals which in turn have made it difficult to obtain the funds necessary to organize large-scale recording operations. Breaking this cycle require concentrated efforts by interdisciplinary teams combining internet and communications technology to develop accessible recording solutions with adequate measurements selected for recording at an acceptable price [32, 33]. From a purely technical perspective most recordings used for breeding evaluations are relatively simple and can be carried out without access to advanced technical infrastructure. Using the Nordic Total Merit Index as an example most measurements (58 out of 83) rely purely on observations and the date of events being recorded (Table 3). A further 15 require a more careful evaluation of the animal prior to an index or evaluation result being recorded. In total only 5 out of 83 traits can be considered difficult to measure from a technical perspective, those traits are related to measurements of milk composition during different lactations [34, 35].

**Table 3:** Measurement types and training requirements for data recording according to Nordic Total Merit Index.

**Measurement type**	**#**	**Training requirements**	**#**
**Recording**	58	Low	39
**Evaluation**	15	Medium	15
**Weighing**	5	High	29
**Chemical analysis**	5	Total number	83
**Total number**	83		

Low training requirements mean that measurements can be done with minimal training, medium with specific training and high requires a trained professional. Very high training is used to signify the need to conduct a full research project prior to obtaining the desired data. For a full breakdown of traits and requirements see supplementary material 2.

In addition developments in the internet and communications technology sector enables farmers to perform more accurate recordings while breeding organizations and advisory organizations can quickly act on the information provided by the farmer and provide value adding services *via* mobile phones [36, 37]. Given the reliance on basic measurements such as the recording of time, volume, weight, height, and behaviour the combination of a high people-to-animal ratio and increasing availability of mobile phones means that individual farmers and community breeding programs can engage in citizen science [38] to provide extensive phenotyping of local genotypes in low and middle-income countries (LMICs). The number of mobile phone users in rural areas has grown rapidly in the previous decade and with on-farm recording systems in place [39, 40] community programs for breeding may thereby provide an important contribution to phenotyping of indigenous livestock populations in LMICs as well as strengthen rural economies [41]. This form of citizen science [42, 43] may therefore become a major contributor to the largescale characterization of phenotypes in indigenous livestock populations as well as direct feedback to the farmers.

### Digitalization of on-farm equipment

A sensor is any device that detects changes in its environment and transmits a resulting impulse. The rapid advancement of information and communication technology mean that devices that detect a change in its environment, convert it to a digital signal and transmit it to a computer processor now make up a large proportion of all sensors. The computer processor then run code which enable information to be analysed, routed or stored as appropriate by the design of the device. Many digital devices are stand-alone systems consisting of one or more sensors, a processor and a display or other interface to interact with the user or other devices. Weather stations used on many farms are a common example of such self-contained devices with a temperature sensor and a humidity sensor attached to a processor which converts the digital signal to numbers which are immediately displayed on a small screen.

Connecting multiple devices into a network requires communication protocols to define how information is received and interpreted when exchanged between components of the network. Large equipment manufacturers such as DeLaval International AB, Lely Industries N.V. and GEA Group AG are transforming their business models into not only providing singular pieces of equipment but also a networked system of devices which together provide the farmer with improved situational awareness, decision support and automation within a proprietary network. From a data collection perspective this means that the availability of data collected from farming equipment is dependent on the relationship between farmers and equipment manufacturers as well as national legalization or adherence to best practice procedures for data ownership and control in a rapidly evolving landscape [44, 45]. Organizations such as the International Committee for Animal Recording (ICAR) also has an important role in the development of this landscape as exemplified by the development of the Animal Data Exchange standard being implemented for dairy through the International Dairy Data Exchange Network which provides a standard and infrastructure for data exchange between machines in the dairy sector [46].

**Figure 2:**
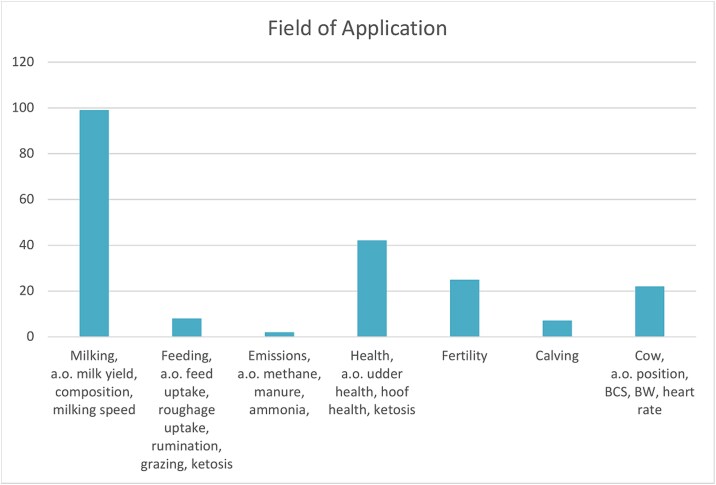
Number of devices capable of recording different traits. Some devices record a large number of different traits meaning that the total number of traits measured is higher than the number of devices in the dataset.

Providing a high-level overview of technology available for phenotyping is complicated by farming equipment being developed for farmers and advisors rather than the scientific community. Publications made by agricultural scientists may cite the agricultural equipment in scholarly papers but the citation standards for equipment are not suited for being readily identifiable in systematic reviews using databases such as Web of Science or Scopus as the Methods & Methods and Introduction field where equipment names and manufacturers are listed are not covered in the database. Google scholar provides better coverage but makes it impossible to define clear inclusion criteria for a systematic review. Thematic surveys and grey literature produced in different projects can however provide valuable snapshots of technology developments and their applications within smart farming [47]. ICAR and the Horizon2020 EU project Data Driven Dairy Decisions for Farmers (4D4F) (Fig. 2) have produced such surveys focused on ruminant animals in 2018 [48, 49] providing an overview of traits and applications of smart farming equipment and sensors. A review of on-farm recording tools for smallholder dairy farming in developing countries was published in 2024 but focused on data curated from scientific literature which likely contributed to the heavy emphasis on recording devices developed by researchers [40].

**Figure 3:**
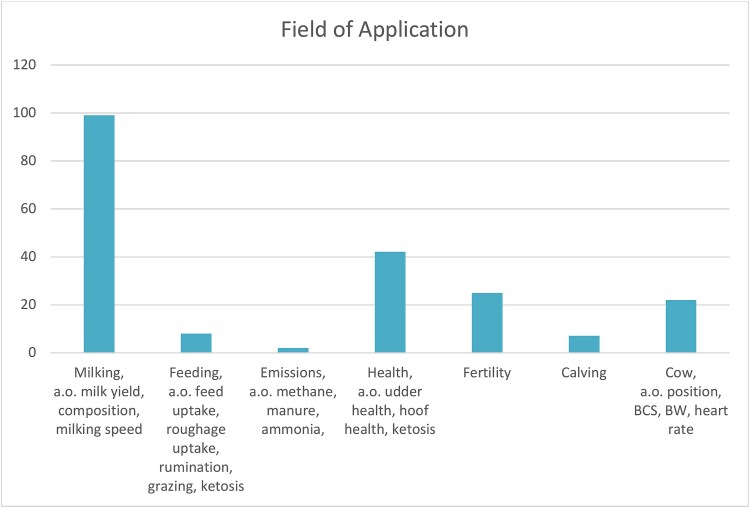
Trait analysis aggregated by field of application.

**Figure 4:**
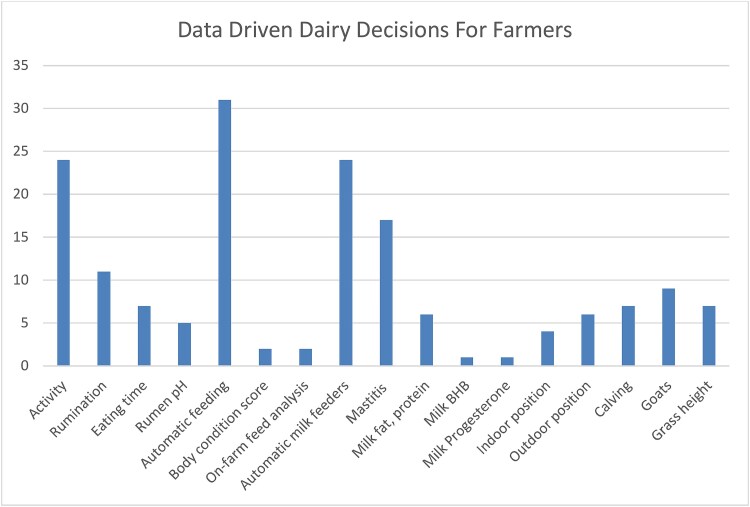
Devices by category included in the 4D4F technology warehouse survey (137 devices out of which some can be used for multiple purposes).

There is a large number of manufacturers with ICAR certified equipment and a majority of the equipment covered by the survey (Fig. 3) [48] are recording milk weight, in the vast majority of cases these machines are also designed to enable sampling of milk for external analysis on accredited laboratories with information also passed on to breeding organizations. In addition many of these machines automatically measure the conductivity of milk which provides early warning of potential mastitis [50]. Looking at the fields of application covered by the study (Fig. 4) milk sensors make up the dominant application of tools (99 out of 155), followed by health measurements (42/155), fertility (25/155), positioning and body condition (22/155), feeding (8/155), calving (7/155), and emissions (2/155).

The 4D4F technology warehouse (https://www.4d4f.eu/content/technology-warehouse) provides an overview of commercial technologies available to monitor and support cow health and performance. This definition includes ‘smart’ equipment such as automatic feeding equipment which may not necessarily generate data for trait recording. In total the warehouse compile information on 137 different devices submitted during the duration of the project [49]. A noticeable difference in this dataset is that a large number of automated feeding devices are recorded but only 7 are registered in the ICAR dataset for trait recording of feed intake. In total 31 devices for automatic feeding, 24 for automatic milk feeding of calves and 7 devices for eating time are listed. Other common areas of utilization for devices identified by 4D4F are activity measurements (24) and mastitis (17) followed by a large number of use areas with a handful of devices in each area.

Restl *et al.* [40] produced a systematic review of on-farm recording systems for smallholder dairy farming. Manual recording using a mobile phone was the dominant form of data recording with only a single Internet of Things (IoT) device (a scale mechanism embedded onto a wheel-barrow to measure milk production of individual cows) was identified in the study. Most recording systems enabled collection of data on milk production (14 out of the 19 systems with reported recording capabilities followed by reproduction (10/19), feeding (6/19), economic performance (3/19 and calf information (2/19).

Looking at the development of equipment covered by these three studies equipment can broadly be categorized into four types of equipment.

- Digitalized farming equipment where embedded systems and new sensors enhance equipment used for example for milking systems, feeding systems and gates which were previously analogue and unable to collect data.
- New wearable devices with sensors.
- Camera and automated monitoring using image analysis.
- Centralized services offered by laboratories and advisory organizations.

### Digitalized farming equipment

Large equipment manufacturers like the aforementioned DeLaval, Lely and GEA have a long history of supplying equipment for farms. Their devices have grown increasingly ‘smart’ by incorporating sensors generating digital input which is processed by embedded computers that control the machine and enables complex operations such as the automated attachment of suction cups to cow teats or control over animal activity by smart gates that provide conditional access to parts of the barn or feed depending on pre-defined rules. The digitalization of equipment also mean that information can be exchanged between different devices on the farm and major equipment manufacturers now offer software branded as farm or herd management systems such as DeLaval DelPro, Lely Time for Cows (T4C) and GEA DairyPlan. These management systems provide farmers with information to support their daily operations.

A single piece of equipment may also host a large number of different sensors to provide information to the farmer and other pieces of equipment as equipment such as milking stations and feeding systems interact with a large number of animals each day, thus making it economically feasible to incorporate devices that would be prohibitively expensive if used to monitor a single animal. As a result, especially milking equipment and associated devices are growing increasingly complex and capable of measuring not only the weight of milk produced but also record values concerning milk composition (fat, protein, and lactose), electrical conductivity, flow rate, peak flow and somatic cell count. Additional measurements of endophenotypes such progesterone to detect heat, lactate dehydrogenase to detect early mastitis, betahydroxybutyrate for ketosis and urea to help farmers adjust the protein intake of animals can also be conducted.

### Wearable devices

The first sensors to measure parameters about single individuals were developed in the 1980s [51]. From a practical perspective, wearable devices can be thought of as one or more sensors, a computer processor, memory and one or more modules for input/output. These devices must be powered and protected from the surrounding environment they are exposed to during the daily life of the animal. A good example and introduction to how a wearable device can be designed integrating multiple sensors and a Bluetooth low energy module with a built-in processor and memory has been published by Pandey *et al.*, [52] documenting how the team developed behavioural monitoring tool for pig farming capturing movement, sound and temperature for processing by machine learning algorithms.

Compared with medical devices, cost control and servicing requirements are much more limiting for farm animal applications, commercially available devices for livestock are therefore largely devoted to measuring physical parameters with most devices focusing on movement and/or temperature which may sometimes be combined with additional information such as sound, pH, or light [48, 49, 51, 53]. These parameters are applicable for a large number of areas and an equipment manufacturer targeting the agricultural sector can thereby purchase generic sensors and other electronics with the livestock-specific component being the physical specifications and the development of algorithms interpreting the data to measure outputs such as activity, heat or calving. The development of biosensors measuring endophenotypes from sweat, blood or other body fluids have been envisaged but have not seen widespread commercialization with the exception of boluses measuring pH in the rumen of cows. The reliance on a pH probe which degrade over time however mean that pH measurements are only available for the first 100–200 days while the bolus remains active measuring temperature and movement in the rumen for up to six years [54].

For livestock there are six common locations for positioning wearable sensor devices, ear, neck, leg, tail, vagina and, for ruminant animals, the rumen. For any sensor there is a trade-off between cost, size, weight, battery life and power usage. The location of the device and size of the animal it is developed for imposes a physical limit to the size of the battery and different manufacturers use different hardware as well as different priorities between the form of the device, sampling intervals and the power of the radio. This mean that for each sensor location there is a range of device with different sensors, battery lives, communication ranges and sampling intervals to choose from, as shown in Table 4.

**Table 4:** A summary of sensor locations, input measurements from sensors, output measurements calculated using algorithms, battery lifetime and range of communications.

**Sensor location**	**Input measurements**	**Output measurements**	**Time**	**Range**	**Intervals**
Ear tag	Accelerometer	Activity, ruminating time, location, heat index, temperature and eating time	2–3 years	200–500 m	15 minutes to every 2 h
	Temperature				
Neck collar	Accelerometer	Activity, ruminating time, location, heat index, temperature, eating time, lying time, standing time and step count.	6 months to 10 years	200–1000 m	Continuous to every 2 h
	Microphone				
	Temperature				
Leg tag	Accelerometer	Activity, standing time, lying time, walking time, step count, heat index (based on movement).	2–10 years	50–1000 m	Continuous to every 2 h
	Temperature				
Tail	Accelerometer		60 days - 5 years	2000 m	
Vagina	Temperature	Calving (tail movement), Calving (temperature), Calving (ejection of sensor)	2 years	1000 m	
	Light				
Bolus sensors	Accelerometer	Activity, temperature, drinking, pH and calving	3–6 years	10–1000 m	15 minutes to every 1 h
	Temperature				
	pH Sensor				

The table is a summary of what is currently achievable among commercially available sensors mounted on different locations of a cow and provides an interval covering the sensors reviewed by Lee *et al.* [47]. The interval ranges from the best in class for each parameter.

**Table 5:** Examples of applications of cameras and imaging methods used to generate data (Fernandes *et al.*).

**Specie**	**Application**	**Image signal**
Cattle	Mastitis	Infrared
	Digital dermatitis	Infrared
	Body temperature	Thermography
	Gait and body measurements	3D
	Weight	3D
	Coat and conformation	Visible light
	Body condition	Visible light, thermography and 3D
Poultry	Behaviour	Visible light and 3D
	Shape	D
Pigs	Tracking	Visible light and 3D
	Behaviour	Visible light and 3D
	Weight	Visible light and 3 D
	Gait and body measurements	3D

### Vision and sound systems in the barn

Fixed mountings of cameras or sound recorders in the barn can provide a cost efficient way of tracking animals, their behaviour and feed intake [55]. For pigs and poultry where the herd sizes are larger and individual value per animal is lower these approaches are especially important as a smaller number of cameras or microphones can be used to maintain surveillance over a large number of animals [56, 57]. Machine learning techniques make it possible to transform non-numeric information such as images into computable data. The emergence of Convolutional Neural Network (CNN) and other deep learning techniques have greatly increase the interest for Computer vision for high-throughput phenotyping in agriculture [58, 59]. When sold commercially many applications are marketed as ‘Artificial intelligence’ or ‘AI’. In practice however each device consists of a recording device connected to an on-board computer or an external server or cloud computing service running a model for classifying data or making predictions based on the feed generated from the recording device. The mathematical theory behind commonly used machine learning is complex and the training of a model for analysis is demanding in terms of data collection, computing power and memory. The popularity of CNNs can at least partially be explained by the increased availability of data combined with CNNs requiring fewer assumptions and less curation of data prior to performing the supervised learning steps to create a prediction model. Multiple toolkits and services are however available to automate or facilitate the process to a level where the process is no more complex than any development of prediction models with supervised learning such as linear regression. As these models can be used on mobile phone cameras or other consumer grade recording devices these models create significant new opportunities for phenotyping at a low cost.

A key challenge working with the commercialization or widespread deployment of camera devices and sound recording systems is the adjustment to different barn environments with different ceiling height, light conditions and equipment obscuring a clear view. Two main approaches are taken to deal with these limitations. Commercial systems like the BCS camera (DeLaval BCS™, DeLaval, Tumba) and Cattle Feed InTake (CFIT) [60] camera have clearly defined installation requirements requiring digital cameras to be installed looking straight down facing a floor or feeding tray with good contrast *versus* the animals or feed measured by the device. Another alternative is transfer learning where a model has been trained on data from multiple sources and location specific training is then performed at a much smaller scale during the installation using a much smaller dataset [61].

Compared to wearable devices the cost of cameras per animal monitored is lower and a review by Oliveira *et al.* [59] show that among a majority of studies done in recent years for using deep learning and computer vision for phenotyping animals pigs were the most common target of research (21/44) followed by dairy cattle (11/44), beef cattle (6/44), poultry (5/11) and goats (1/44). For applications where cameras have been tested visible light cameras providing imagery in 2D or combined with some kind of depth sensor are the most common as they enable animals to be tracked looking for abnormal behaviour, activities, social interactions and locomotive issues such as lameness. Infrared cameras have also been used either to provide better data in low-light conditions or as a way to measure abnormal temperatures on parts of the body which may indicate mastitis or digital dermatitis. Numerous detailed reviews on these applications and the quality of data extracted from imagery using different algorithms have been published in recent years [17, 58, 59].

### Centralized services

Breeding organizations and advisors often collaborate with laboratories, slaughter houses and other service organizations to obtain data. Laboratory milk analysis, conformation recording, animal identification, genomic services and carcass evaluations are examples of services often provided in this way. In some cases, it would be difficult to replicate the services elsewhere, in others, digitalization, automation and miniaturization mean innovations in wearable device or digital farm equipment may supersede or replace centralized services by putting them closer to the farmer. As early as 2006, experts analysed the potential impact of the small but growing use of on-farm decision-support systems and how automated on-farm recording systems would impact the collection of data for breeding programs [62]. Poor data quality caused by a lack of machine calibration, a lack of communication between on-farm systems and Dairy Herd Improvement Agencies and the risk of abundant low-quality information crowding out more valuable information were highlighted as key challenges for future breeding programs.

Simultaneously the employment of specialists working with advanced tools and incentives to remain in business create incentives and opportunities for new services to be developed by service providers. Utilizing artificial intelligence to automatically analyse animal-based welfare indicators in abattoirs have been proposed as a more cost-effective and objective method for welfare surveillance in swine production compared with on-farm evaluations [63]. In a similar vein automated image analysis in abattoirs have been proposed as a more objective way of grading muscle size, tenderness, intramuscular fat and marbling in beef [64], pork [65] and lamb production [66].

Laboratory services for milk testing using Mid-infrared spectroscopy (MIRS) showcase the dynamic relationship between on-farm testing and centralized services. MIRS function by measuring the proportion of infrared light being absorbed by different molecular bonds in the sample. Using different mathematical techniques and signal processing this creates a spectrum with absorption as a function of wave lengths between 2500 and 25,000 nm which can be used to estimate the concentration of key components in the milk sample [67]. MIRS can be used for a wide variety of applications but many agricultural organizations have sub-contracted service providers for analysis of milk samples to report the computed measurements of key components such as fat, protein and somatic cell count without storage of the full spectral data [68]. This limited set of services can now be performed on farms using modern milking equipment and machines leading to the challenges described by Wade in 2006 [62]. Initiatives such as OptiMIR, HappyMoo [69] and D4Dairy [70] have however combined full spectral data with machine learning techniques and on-farm data to greatly expand what can be measured using MIRS. Biomarkers so far tested with MIRS include markers for energy deficit energy deficit (citrate, isocitrate, glucose-6 phosphate [glucose-6P], free glucose), ketosis (β-hydroxybutyrate and acetone), mastitis (N-acetyl-β-d-glucosaminidase activity and lactate dehydrogenase), and fertility (progesterone) [71], in addition research indicate that predictions from MIRS data may also be capable of replacing direct measurements of functional traits difficult to measure like nitrogen efficiency [72] and methane gas emissions [73].

In addition to data collection of full milk spectra, the successful estimation of endophenotypes or functional traits from MIRS requires large datasets for technical standardization, calibration and selection of a prediction model suited to the biomarker being measured [67, 69, 70, 74]. Given the current emphasis on “AI models it is also worth noting that simpler more interpretable models like partial least squares regression, or least absolute shrinkage and selection operator may equal or outperform the predictions of neutral networks [74]. The costs of operating a lab able to routinely produce high quality MIRS data and the need to calibrate prediction models for each endophenotype being studied mean that the start-up costs for any new phenotype measured in a population using MIRS will be high but any such investment will also see a high degree of scalability once validated and implemented.

### Contextual phenotyping and remote monitoring

Phenotype plasticity is the ability of an organism to change in response to stimuli or inputs from the environment. Phenotypic plasticity can be a source of ‘noise’, or confounding variation in experiments [9]. In livestock farming however, phenotypic plasticity is often in itself a trait of relevance to farmers and breeders as traits such as resilience are desirable and heritable characteristics of an animal [75]. Heritability of traits as measured by breeders is also dependent on animal environment interactions leading to decreased heritability estimates of traits for commercial livestock when imported from temperate climates to the tropics [76]. These challenges are especially clear in extensive production systems where animals to a greater degree are dependent on their local environment [77]. For researchers integration of data may help researchers in both applied agricultural sciences and more fundamental genetics research regarding environmental interactions to contextualize phenotype data and test the variance and covariance structure under a wider range of conditions, ideally using continuous environmental gradients [78]. With sufficiently large datasets researchers can thereafter start untangling correlated phenotypes, separating phenotypes such as successful grazing behaviour *versus* high metabolic efficiency or resilience towards parasites.

Much work is still needed on developing frameworks to formulate data-driven questions and identifying suitable research environments where information about animals, herds, farming operations and local environment can be merged to capture phenotype plasticity [21]. This work does not only require the integration of new sensor technologies but also the selection of algorithms suited for different classification and prediction tasks. Machine Learning methods are widely used in genetics [79] and described further in the phenotyping methods section of this review. As datasets become increasingly complex and reliant on multiple data sources a shift from regression-based methods to methods such as Random Forest or Neural networks can improve the scalability of projects by requiring less pre-processing and assumptions when selecting a regression model. Kamphuis *et al.* recently published a study exemplifying this by comparing logistic regression and random forest for predicting lifetime resilience scores [75]. From a methods development perspective, the study showed random forest to only provide slightly better classification performance but that the method is more scalable for large scale project as data requires less pre-processing and optimization to achieve this performance. The study therefor does not only exemplify the value of data integration from multiple sensors such as surveillance drones with automated localizations, identification and activity, but also how algorithms for data analysis must not only be evaluated on performance but also scalability and ease of use [75].

Key technologies suitable for providing contextual information on pastures have recently been summarized by Herlin *et al*. [80] highlighting that even though the development of sensors for livestock primarily have targeted indoor usage there is rapid development of new technology for outdoor usage as well. Overall the combination of improved batteries, development of drones as a platform for video cameras and access to electronic positioning and transfer systems including RFID, Wireless Sensor Networks (WSN), Global Positioning (GPS), the IoT, and Low-Power Wide-Area solutions enables farmers, advisors, and researchers unprecedented access to data concerning the environment and activities of animals on pastures. Of special note is that when optical identification and tracking is possible positioning can be as accurate as 1–3 m [81] while GPS collars are limited to an accuracy of 7–13 m in open terrain while providing robust but less exact positioning measurements 19–30 m) in dense forests [80].

In addition to sensors tracking animals, remote measurement techniques using satellites to monitor crops, land use and livestock movements have taken an important role in precision agriculture for pasture monitoring and crops production. Services like Cropsat (https://cropsat.com/) using satellite imagery from the European Sentinel-2 satellites are now freely available on line with resolutions down to 10 × 10 m to 60 × 60 m resolution depending on the wavelength [82]. These data can be used to evaluate biomass growth, surface water coverage for land surveillance and also specifically used for estimations for pasture quality [83]. Researchers affiliated with International Livestock Research Institute have developed one of the first practical applications for satellite imaging being used for pastoral farming in LMICs. An Index-Based Livestock Insurance program shows that satellite data can provide an early and robust warning of adverse growth conditions for livestock [84] giving farmers improved financial flexibility to deal with adverse conditions. This example demonstrates how the integration of satellite data used for economic purposes to deal with environmental challenges, if appropriately measured, may turn out to be a long-term value for phenotyping and research on livestock. Similar models may be applied not only for assessing the context of measurement to study characteristics such as resilience of animals [75] but also to provide an opportunity for farmers to be compensated for environmentally friendly activities taken to ensure biological diversity or wildlife friendly farming [85].

## Conclusion

Livestock farming and breeding is dependent on large scale data collection and analysis. Within the sector, new wearable devices, networked services and low cost information and communications technology make data collection cheaper and more accessible than ever before. New implementations of AI for analysis of images, video feeds or sound recordings also make it possible to collect data from individual data in species where the low economic value and physical characteristics of an individual otherwise would make individual data collection difficult. Through collaboration with the agricultural sector, genetics researchers can hereby obtain data from thousands of animals at a fraction of the cost for raising animals specifically for research. In some cases, this data collection is enabled by high-end technology requiring significant capital investments in equipment such as dairy milking robots or activity sensors. In many cases the key to successful phenotyping however lies in the organization of farmers, breeding organizations and advisors to collect relevant information. Internet access and mobile phones here serve as key enablers to make recording schemes viable even in low income settings even if much work is still limited to researcher-dominated projects.

Data collection and aggregation also make up a second space for AI implementations. Decision support systems rely on aggregation of data from a large number of data sources and their implementation in precision livestock farming. Combined with the growing interest in the environmental component in livestock farming this creates further opportunities for researchers to access data for phenotyping. Weather data and environmental monitoring from programmes such as the EU Sentinel 2 satellites here make it possible to better study the impact of environmental factors as well as gene–environment interactions over long periods of time. Taken together this mean that livestock stock farming is a sector which is well positioned to support large-scale data collection efforts and use the data both for commercial interests and as a contribution to fundamental genetics researching exploring complex traits and the complex interplay between genes and environment.

**Key points of the article**

- As genetic data becomes increasingly accessible, phenotyping replaces genotyping as the bottleneck in genetics research.
- Economies of scale mean that when performed in collaboration with the industry chips such as 45 k EuroG MD beadchip for cattle can be used to genotype animals for a price of <30 € per animal.
- Data collection from farm animals benefit from digitalization of farming equipment. New wearable devices and devices such as cameras or audio recorders using machine learning (often refered to as ‘AI’) to generate physical measurements and activity data are becoming increasingly common.
- Contextual information is becoming increasingly available as large open data sets measuring weather, biomass and other environmental factors become available as a part of the digital transformation of society as a whole. Advanced data processing has been a major part of agriculture for over a century and in this context the emphasis on ‘AI’ can serve to rapidly expand the toolset and data sources to augment existing industry infrastructures supporting high-throughput collection of data which can be used for phenotyping.
- Even with digitalization most traits used in current breeding programs are still of a simple nature. Combined with information and communication technologies accessible through cheap telephones. This creates an impetus for high-throughput phenotyping in collaboration with smallholder farmers in low- or middle-income countries that are hotspots for genetic diversity due to the preservation of indigenous breeds.

## Author contributions

Tomas Klingström (Conceptualization [equal], Data curation [lead], Formal analysis [lead], Methodology [lead,] Visualization [equal], Writing – original draft [lead], Writing – review & editing [equal]), Emelie Zonabend Konig (Conceptualization [lead], Funding acquisition [equal], Investigation [equal], Methodology [equal], Validation [equal], Writing – original draft [supporting], Writing – review & editing [equal]), Avhashoni Zwane (Conceptualization [equal], Formal analysis-Supporting, Investigation [supporting], Methodology [supporting], Validation [equal], Writing – review & editing [equal])

Conflict of interest: None declared.

## Funding

Funding was received through the Livestock Genetics Flagship of the Livestock CGIAR Research Program. Work was performed as part of a South Africa (NRF)/Sweden (STINT) science and technology research collaboration (SA2018-7728) with additional funding from Kunskapsnavet för jordbrukets digitalisering which is partially funded by the European Agricultural Fund for Rural Development.

## Supplementary Material

Supplementary_Material_1_elae032

SupplementaryMaterials_2_elae032

## Notes

**Tomas Klingström**, PhD is the coordinator of the SLU Infrastructure for dairy data collection, Gigacow. His research work is mainly focused on using agricultural technology for high-throughput phenotyping of livestock.

**Emelie Zonabend König**, PhD is a research advisor at SLU Global, at SLU’s Vice Chancellor’s office and an animal breeding specialist. In her research she is focusing on animal breeding strategies in low- and middle income countries.

**Avhashoni Zwane**, PhD, is a genomic specialist whose work focus on marker and gene discovery in livestock animals, as well as mapping of genes for disease resistance against fungal pathogens in plants.
